# Dance/Movement Therapy as an Intervention in Breast Cancer Patients: A Systematic Review

**DOI:** 10.1155/2021/4989282

**Published:** 2021-11-23

**Authors:** Natalja Fatkulina, Vaiva Hendrixson, Alona Rauckiene-Michealsson, Justina Kievisiene, Arturas Razbadauskas, Cesar Agostinis Sobrinho

**Affiliations:** ^1^Faculty of Medicine, Vilnius University, Vilnius, Lithuania; ^2^Faculty of Health Sciences, Klaipeda University, Klaipeda, Lithuania

## Abstract

**Aim:**

In this paper, we systematically review the evidence looking at the effect of dance/movement therapy (DMT) and mental health outcomes and quality of life in breast cancer patients.

**Method:**

The literature search was done with the databases PubMed (MEDLINE), EBSCO, and Cochrane Central by using the following search words: “dancing/dance/movement therapy,” “breast cancer/neoplasms/carcinoma/tumour” or “mammary cancer,” “mental health,” and “quality of life.” Ninety-four articles were found. Only empirical interventional studies (*N* = 6) were selected for the review: randomised controlled trials (RCT) (*n* = 5) and non-RCT (*n* = 1). PRISMA guidelines were used.

**Results:**

Data from 6 studies including 385 participants who had been diagnosed with breast cancer, were of an average age of 55.7 years, and had participated in DMT programmes for 3–24 weeks were analysed. In each study, the main outcomes that were measured were quality of life, physical activity, stress, and emotional and social well-being. Different questionnaires were used for the evaluation of outcomes. The mental health of the participants who received DMT intervention improved: they reported a better quality of life and decreased stress, symptoms, and fatigue.

**Conclusion:**

We found only six studies for review, and some had a small number of participants. However, our findings indicate that DMT could be successfully used as a complimentary therapy in addition to standard cancer treatment for improving the quality of life and mental health of women who have been diagnosed with breast cancer. More research is needed to evaluate the complexity of the impact of complimentary therapies. It is possible that DMT could be more effective if used with other therapies.

## 1. Introduction

As a method of treatment, dance/movement therapy (DMT) belongs to the category of complimentary or alternative medicine [1]. Since dance is a type of physical activity, some authors have proposed that it should be used as an additional therapy for women with breast cancer to achieve some health benefits [2, 3]. DMT explores awareness, expression, and acceptance of the body, which can improve physical, emotional, and cognitive integration [4]. It has also been shown that dancing can promote the sharing of feelings and helps to reduce the loneliness and isolation of patients [3, 5].

The American Dance Therapy Association (ADTA) defines DMT as a multidimensional approach that integrates body awareness, creative expression, and the psychotherapeutic use of movement to promote the emotional, social, cognitive, and physical integration of the individual to improve health and well-being [6]. The European Association of Dance Movement Therapy adds “spiritual integration” to this list [7]. Thus, it seems that DMT could be an effective solution for many patients with various diagnoses and their partners. However, patients with breast cancer are one of the most vulnerable groups with which DMT is used.

Breast cancer is the most frequently diagnosed cancer among women worldwide [8–10]. Approximately 13% of women (1 in 8) will be diagnosed with invasive breast cancer in their lifetime [9]. Lifetime risk reflects an average woman's risk accounting for deaths from other causes that may pre-empt a diagnosis of breast cancer [11]. Breast cancer rates have fallen steadily, with about a 35% decline in rates over the past three decades [10]. This is likely due to reduced hormone replacement therapy use, improvements in screening, early diagnosis, and treatment [11]. Because of population ageing, however, the number of breast cancer deaths is not declining [9].

Even though survival rates are continually increasing, breast cancer is often associated with long-term psychological distress, chronic pain, fatigue, and impaired quality of life [8]. Many women who currently are undergoing treatment or have completed treatment for breast cancer use complementary therapies to manage the effects of the disease [8, 12]. As a mind-body integrated form of psychotherapy, DMT combines the benefits of dance, movement, emotional expression, social support, and creative activity in a single intervention [13].

How DMT can be used to improve psychological and physical outcomes in breast cancer patients has been evaluated in previous reviews [13–15]. Their results showed that DMT had positive effects on physical health, mental health, and quality of life in general [13–15].

The aim of our study is to determine from interventional experimental studies whether DMT can improve the quality of life, mental health, and symptoms related to cancer in women with a diagnosis of breast cancer and whether DMT is an appropriate method of complementary medicine to improve the general condition of patients.

## 2. Methods

**Figure 1:**
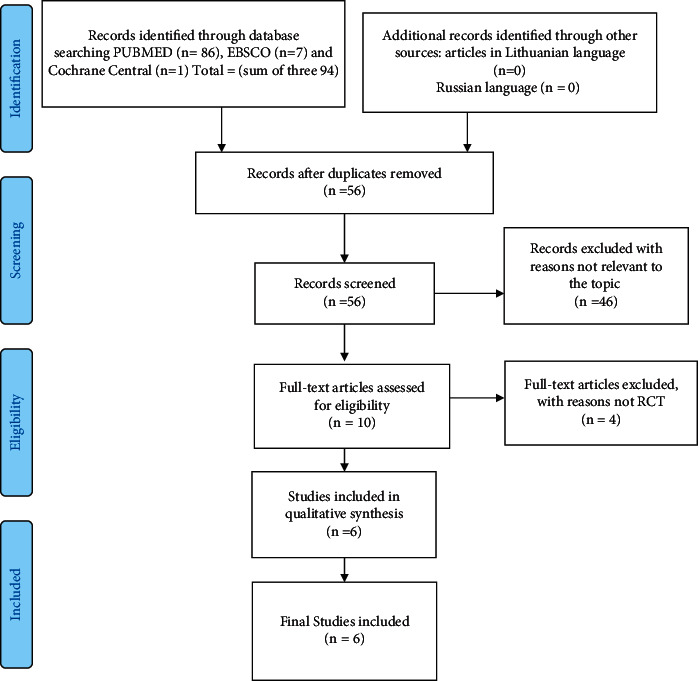
Search and selection process.

A systematic literature review using PRISMA guidelines was done (see Figure 1). The search for literature in an electronic format was done in the databases PubMed (MEDLINE), EBSCO, and Cochrane Central by using the following terms: “dancing/dance/movement therapy,” “breast cancer/neoplasms/carcinoma/tumour,” “mammary cancer,” “mental health,” and “quality of life.” Ninety-four articles were found. Additional searches were done by using the same search words in the Lithuanian and Russian languages, but not one study was found. Six empirical interventional studies reported in English language, full text and published from January 2005 to February 2020, were selected for the review: RCT (*n* = 5) and non-RCT (*n* = 1). All studies included DMT interventions and outcomes for women with breast cancer. Data were analysed by using content analysis with PRISMA guidelines [16, 17]. There were no ethical problems to report during the search, selection, or data analysis processes.

## 3. Results

### 3.1. Location of Studies/Geography

The interventional experimental studies about the impact of DMT were conducted in various countries and continents: in Europe from Greece and Portugal [2, 3]; in Asia two from Hong Kong, but they were done by the same team in different years [18, 19]; in North America two from the USA [20, 21]; and in South America from Brazil [3], see Table 1.

### 3.2. Participants in Studies

The identified participants in this literature review were women who had been diagnosed with breast cancer and who agreed to participate in the research (*n* = 385). Their total average age was 55.7 years. In one study [21], partners of survivors also participated in the study (*n* = 31). In all studies, two groups were investigated: an experimental group that took part in DMT and a control group that did not take part in a DMT programme. The groups were similar in all parameters.

### 3.3. Intervention Programmes

The interventions for the experimental groups were dance therapy, movement therapy, or dance and movement therapy (all interventions were investigated as DMT), but all were specifically designed to meet the abilities of breast cancer survivors. Only qualified specialists who had extensive experience in fitness dancing rehabilitation such as physical education teachers, physical therapists, and experienced dance instructors were responsible for the sessions and quality of DMT intervention during the intervention time. All programmes were specially adapted or designed for the participants in the experimental group. Programmes included check-in, warm-up, stretching, main training and dancing/movement, upper body training, and relaxation. The sessions were conducted in small groups or privately. The duration of the programme differed from 3 weeks [18, 19] to 24 weeks [2], with the most used duration being 12 weeks [3, 20, 21].

Additional instruments were used in the studies for physical evaluation: measurement of blood pressure, heart rate, and various physical examinations. In all studies, various questionnaires were used for evaluation of mental health aspects. The Godin Leisure Time Exercise Questionnaire, International Physical Activity Questionnaire, and functional capacity walk tests were used. To evaluate participants' psychological condition, the Beck Depression Inventory (2 times), Life Satisfaction Inventory, Quality of Life-Breast Cancer, Global Quality of Life, Symptom Bother Scale, Body Image, Hospital Anxiety and Depression Scale (2 times), Fear of Recurrence Scale, Mindful Attention Awareness Scale, Self-Compassion Scale, Five Facet Mindfulness Questionnaire, Experiences Questionnaire, Daily diary, Perceived Stress Scale (2 times), Brief Fatigue Inventory, Brief Pain Inventory, Pittsburgh Sleep Quality Index, Functional Assessment of Cancer Therapy-Breast Scale, Quality of Life SF-36, Dyadic Trust Scale, Dyadic Adjustment Scale, Quality of Life Questionnaire C30–EORTC QlQ-C30, and Piper Fatigue Scale.

### 3.4. The Main Findings

In all studies, the main outcomes that were measured were quality of life, physical activity, stress, and emotional and social well-being. All studies showed the positive effect of DMT on outcomes. In the evaluation of the physical parameters of the DMT experimental and control groups, some positive changes were discovered in the DMT group. Quality of life improved after DMT, and fatigue, depression, stress, and anxiety decreased. Pain was not investigated as an outcome in any of the studies.

## 4. Discussion

The present systematic review summarises the evidence from interventional experimental studies concerning DMT on quality of life, mental health, and symptoms related to cancer in women who have been diagnosed with breast cancer. Overall, the results showed that DMT had positive effects on physical health, mental health, and quality of life. Our results are consistent with prior reports [13–15]. Our study is useful and has value because of analysing RCTs on a specific group of patients with breast cancer who need specific health care.

DMT, a movement-based therapy involving mental health issues and artistic components, showed that it had the ability to reduce stress [19]. Breast cancer mortality could be reduced by using high-quality prevention and high-quality interventions such as DMT [11]. It is necessary to have a high level of knowledge in the field to focus on disadvantaged groups [10]. All studies involved in the review showed that complimentary therapies can significantly influence recovery and rehabilitation of breast cancer patients.

The studies presented in this systematic review have different methodologies, results, and interventions. However, the main finding was common between them, showing that DMT has a positive impact on body recognition and mental health status. Moreover, the authors state that these effects seem to be because of the combined benefits of dancing/moving, emotional expression, and psychosocial support [13–15].

In some studies, the DMT was a mix of activities such as a combination of aerobic exercises with Greek traditional dances [2] or special programmes that included stretching, relaxation, rhythmic body movement, and improvisational dance [18, 19]. In other studies, special DMT programmes were conducted: the Mindful Movement Program [20], light intensity ballroom dancing [21], and belly dancing [3]. Thus, all types of DMT seem to have important health-related outcomes for patients, but the combination of movement and relaxation may have a more positive effect on quality of life.

The average age of the participants in the studies varied, with the youngest group from Hong Kong at 49 years [18, 19] and the oldest from USA at 66 years [20]. The numbers of participants also differ from 8 in the intervention group and 11 in the control group [3] to 69 in the intervention group and 70 in the control group [18]. In other studies, the numbers of participants involved in the experimental and control groups were the following: 14 and 13 [2], 29 and 19 [20], 15 and 16 [21], and 63 and 58 [19]. Future studies must involve more participants to provide more comprehensive and accurate information and knowledge regarding the impact of DMT on patient health and well-being.

The type and duration of the intervention also differ between the studies. The longest one included a Greek traditional dancing programme and aerobic training [2] and lasted 24 weeks (3 times per week, 60 minutes per session). The shortest one was a special programme including stretching, relaxation, rhythmic body movement, and improvisational dance [18, 19], which lasted 3 weeks (6 sessions, twice a week, 1.5 hours per session). The duration of other studies with DMT intervention was between 10 and 12 weeks [3, 20, 21]. With the aforementioned information in mind, it seems that the optimal duration and frequency for this treatment could be about 12 weeks (3 months), with a DMT session 2 or 3 times per week.

Quality of life was measured as the main outcome in all studies. Other outcomes that were investigated were physical function/activity and physical well-being [2, 3, 20, 21]; mental health, including psychological, emotional, and spiritual well-being [2, 20, 21]; stress, anxiety, fatigue, and depression [2, 3, 18, 19]; pain and sleep disturbance [18, 19]; social well-being [20]; and vitality, dyadic trust, and relationship with partners [21]. Overall, the main findings showed that, in all studies, the DMT interventions were effective and had a positive impact on quality of life, with improvement in both physical and mental health. Moreover, the participants' relationship with their partner became better after DMT sessions.

DMT also has an impact on the behaviour of patients. Behavioural engagement is built into the treatment, which is structured to help patients to explore, try, and learn new ways of communication [13]. Specifically, studies on DMT have indicated its efficacy as a complementary and holistic intervention in providing social support, decreasing fatigue and stress, increasing mobility, and enhancing the overall well-being of cancer survivors [22]. DMT could be beneficial for women with breast cancer in some ways: helping to cope with treatment and physical symptoms, improving mental health and appreciation for self and body, improving total functioning, and aiding faster recovery back to a better life [19].

Therefore, according to our findings in this systematic review, DMT has been shown to improve the health of women with breast cancer in connection with their mental health: (1) physical condition and activities, including body image and body recognition; (2) quality of life and well-being; and (3) stress, depression, fatigue, and anxiety. We did not find any positive impact on pain or pain management.

## 5. Conclusion

DMT as an integrated form of psychotherapy can improve the physical and mental condition of patients with breast cancer. Overall, our findings indicate that as a complimentary therapy, DMT can be successfully used in addition to standard cancer treatment to improve the quality of life and mental health of women who have been diagnosed with breast cancer and their families. Moreover, DMT could be very beneficial in the treatment of breast cancer, the main purpose being to improve the patient's general condition and to help the patient to achieve a better mental health and better quality of life. More research is however needed to evaluate and investigate the complexity of the impact of DMT and other complimentary therapies such as art therapy and music therapy on the mental health of breast cancer survivors.

## Figures and Tables

**Table 1:** Reviewed studies in dance/movement therapy according to sample, method, outcomes, and main findings.

Author (year)	Country	Randomized controlled trials (RCTs)	Population, sample, and age	Intervention and duration	Outcomes	Main findings
Kaltsatou et al. (2011)	Greece	Yes	*N* = 27 (DMT *n* = 14, 56.6 years, control *n* = 13, 57.1 years)	Greek traditional dancing programme and aerobic training 24 weeks (3 times per week, 60 min DMT session)	Quality of life, physical function, emotional well-being, and depression	Combined exercise program (aerobic exercise with Greek traditional dances) has beneficial effect in physical function, life satisfaction, and depressive symptoms

Crane-Okada et al. (2012)	USA	Yes	*N* = 48 (DMT *n* = 29, 66.1 years, control *n* = 19, 64.8 years)	Mindful movement program 12 weeks (daily)	Quality of life (psychological, physical, spiritual, and social well-being) Mindfulness (attention, intention, and attitude)	Preliminary evidence for feasibility of the intervention was demonstrated in the areas of acceptability, demand, implementation, practicality, and limited efficacy

Rainbow Ho et al. (2016)	Hong Kong	Yes	*N* = 139 (DMT *n* = 69, 48.6 years, control *n* = 70, 49.1 years)	Special programme, including stretching, relaxation exercises, movement games, and rhythmic body movement 3 weeks (twice a week, 1.5 hour DMT session)	Stress, anxiety, depression, fatigue, pain, sleep disturbance, and quality of life	The short-term DMT program can counter the anticipated worsening of stress and pain

Rainbow Ho et al. (2016)	Hong Kong	Yes	*N* = 121 (DMT *n* = 63 49.1 years, control *n* = 58 49.8 years)	Special programme, including stretching, relaxation, rhythmic body movement, and improvisational dance 3 weeks (6 sessions, twice a week, 1.5 hours)	Stress, fatigue, pain, and sleep disturbance, quality of life	DMT might have a beneficial effect on diurnal cortisol slopes in breast cancer patients with high levels of distress

Pisu et al. (2017)	USA	Yes	*N* = 31 (DMT *n* = 15, 56.7 years, control *n* = 16, 59 years)	Movement (RHYTHM) project: ballroom dancing programme 12 weeks (10 private lessons, 45 min weekly dance, and 2 group lessons/practice parties)	Quality of life, physical activity, mental health, vitality, dyadic trust, and relationship with partners	Light intensity ballroom dance may be an important tool to return to a physically active state and improve quality of life and other aspects of intimate life

Boing et al. (2018)	Brazil, Portugal	Non-RCTs	*N* = 19 (DMT *n* = 8, age not indicated, control *n* = 11, age not indicated)	Belly dance programme 12 weeks (twice a week, 60 min DMT session)	Quality of life, fatigue, depressive symptoms, and physical activity	Belly dance can be a viable form of physical activity, improves quality of life, and decreases fatigue and depressive symptoms

## Data Availability

The data used to support the findings of this study are included within the article.
